# Expressional Analysis and Role of Calcium Regulated Kinases in Abiotic Stress Signaling

**DOI:** 10.2174/138920210790217981

**Published:** 2010-03

**Authors:** Ritika Das, Girdhar K Pandey

**Affiliations:** Department of Plant Molecular Biology, University of Delhi South Campus, Benito Juarez Road, New Delhi-110021, India

**Keywords:** Calcium, abiotic stress, kinase, CBL, CIPK, CDPK, CaM, signal transduction.

## Abstract

Perception of stimuli and activation of a signaling cascade is an intrinsic characteristic feature of all living organisms. Till date, several signaling pathways have been elucidated that are involved in multiple facets of growth and development of an organism. Exposure to unfavorable stimuli or stress condition activates different signaling cascades in both plants and animal. Being sessile, plants cannot move away from an unfavorable condition, and hence activate the molecular machinery to cope up or adjust against that particular stress condition. In plants, role of calcium as second messenger has been studied in detail in both abiotic and biotic stress signaling. Several calcium sensor proteins such as calmodulin (CaM), calcium dependent protein kinases (CDPK) and calcinuerin B-like (CBL) were discovered to play a crucial role in abiotic stress signaling in plants. Unlike CDPK, CBL and CaM are calcium-binding proteins, which do not have any protein kinase enzyme activity and interact with a target protein kinase termed as CBL-interacting protein kinase (CIPK) and CaM kinases respectively. Genome sequence analysis of Arabidopsis and rice has led to the identification of multigene familes of these calcium signaling protein kinases. Individual and global gene expression analysis of these protein kinase family members has been analyzed under several developmental and different abiotic stress conditions. In this review, we are trying to overview and emphasize the expressional analysis of calcium signaling protein kinases under different abiotic stress and developmental stages, and linking the expression to possible function for these kinases.

## INTRODUCTION

Plants are constantly challenged in an environment by several stimuli and factors, which guide in regulating physiological and developmental programs. Plants need to sense and process the environmental stimuli such as light, temperature, water status, and nutrient availability, in order to grow and develop normally, but under adverse environmental condition, several signals restrict the normal growth and development. There are two major classes of stresses, namely, abiotic and biotic, which can hinder and threaten plant viability. 

Agronomically, environmental stresses are the major cause of loss of crop productivity worldwide and pose a basic threat to the plant ecosystem as a whole. Therefore, it is of immense importance to understand the molecular and biochemical basis of how plants perceive and combat environmental stresses. In response to these adverse signals, plants activate their cellular machinery comprising of a plethora of proteins which are involved in perception, signal transduction and respond to these stimuli that help plants to adjust or adapt to these adverse conditions, and which ultimately leads to tolerance or survival of the plants. Signal transduction pathway comprises of multiple arrays of proteins, especially protein kinases, which are involved in the phosphorylation-mediated pathway, and play a crucial role for plants to tackle the environmental stresses they encounter.

Calcium serves as ubiquitous and a central hub in a large number of signaling pathways. Multiple extracellular signals such as light, hormones, biotic and abiotic stimuli elicit changes in calcium levels in the cell [1-4]. Biologists were fascinated at how several different stimuli or stress signal leads to changes in the calcium concentration, and how specificity in signaling network is being maintained. A number of studies in animal and plants have already proven the fact that calcium signal is not only represented as changes in intracellular concentration but also as spatial and temporal information that is also encoded along with [5-7]. In addition, a terminology called “Calcium Signature” has been coined for these spatial and temporal changes in calcium concentration, which generates specificity in calcium-mediated signaling pathway [5-7]. Alterations in the level of calcium is an event that triggers a whole range of signal transduction pathways *via*  different calcium sensors that are either induced or are already present in the cell. All the eukaryotic cells have a multiple number of calcium sensors, which are responsible for detecting the changes in calcium concentration. Therefore, a very precise level of regulation is being mediated by these calcium binding proteins which act as sensors of calcium [8-12]. In plants, calcium sensor proteins are categorized as calcium sensor responder and sensor relay [10]. The calcium relay protein bind calcium and affect their target protein since they themselves do not have enzymatic activity, and typical example of these are calmodulin (CaM) and calcineurin B-like (CBL) protein [9]. In contrast, the sensor responder proteins bind calcium and a change in conformation takes place, and hence modulates their own activity by intra-molecular interaction [13]. Calcium dependent protein kinases (CDPKs) are the best characterized protein family, which has calmodulin-like calcium binding domain and a Ser/Thr protein kinase domain in a single protein. Upon binding calcium, they are directly activated and transduce the signal *via*  phosphorylation cascades and regulation of gene expression [1, 9, 14-16]. CaM and calmodulin-like protein have been best characterized as a calcium sensor relay, which do not have enzymatic activity, and hence activate or deactivate their interacting proteins [9]. A new family of calcium sensors from Arabidopsis has been identified, which is similar to calcineurin B-subunit and neuronal calcium sensor from animals [9, 17, 18]. These plant calcium sensors were referred as calcineurin B-like (CBL) proteins [18]. Like calmodulin, CBL proteins are calcium sensor relay, which upon binding calcium undergo changes in conformation and activate their target proteins. CBL protein interacts with a novel SNF1-like protein kinase family called CIPK [9, 12, 19-22]. Besides these well characterized kinases involved in signal transduction pathways, there are several other calcium regulated kinases such as Ca^2+^/CaM kinase (CCaMK) and CDPK-related protein kinase (CRK) that are responsive to stress, and also exhibit development specific expression pattern [23]. However, their exact nature and role in stress signaling requires detailed investigation.

In this review, we are trying to focus on expressional analysis of these calcium signaling kinases emphasizing on the functional regulation by expression profiling which combined with functional analysis of these different calcium-signaling kinases under diverse stress conditions, development and stage specific studies will be overviewed. Moreover, the expression data together with cellular localization and protein-protein interaction will also be touched upon for some of these kinases in activating signaling cascades upon sensing environmental stress.

## CALCIUM DEPENDENT PROTEIN KINASES (CDPKS) AND ABIOTIC STRESS

Calcium dependent protein kinases or CDPKs that are the most extensively studied calcium signaling kinases act in a manner which is independent of upstream sensors since they directly bind and sense calcium ions to activate the downstream signaling in response to different stress and development cues [14,15, 24]. CDPKs have been found in a wide range of plant species and in some of the protists [15]. Other eukaryotic genomes such as that of yeast and other animals have not shown the presence of CDPKs [14]. CDPKs are predicted to be having molecular weight between 54.3 kD to 72.2 kD with the differences in variable domains mainly contributing to this size difference [14]. N-terminal domain possesses potential myristoylation (Glycine residues) and palmitoylation sites (Cysteine residues), which might be important for membrane association as demonstrated by various studies [25, 26]. Such membrane associations are important for sensing the changes in calcium fluxes, which occur mostly at these membrane sites. An adjacent junction domain follows the kinase domain, which is near to the N-terminal, which is inhibitory in function. Next to this domain lies the calmodulin-like, calcium-binding domain which comprises of four EF-hands responsible for binding to the calcium ions. The kinase activity of the CDPKs has been confirmed not only by the identification of physiological substrates that are shown to be phosphorylated by CDPKs [27], but also using cell free systems where synthetic peptides used as substrates were shown to be phosphorylated in presence of calcium by CDPKs translated from cloned CDPKs using wheat germ extract [28]. Of the twenty six Arabidopsis CDPKs studied, seven were found to be showing both autophosphorylation as well as phosphorylation of the biotinylated peptide at the threonine residue. In addition to calcium ions, phosphorylation, membrane phospholipids and 14-3-3 proteins are also found to be positively regulating the activity of different CDPK proteins [29]. Besides showing up regulation under different stress conditions, significant numbers of reports have also indicated the role of CDPKs in hormonal response, regulation by light and pathogen attack [30-32]. With respect to tissue specific expression, it was seen that most CDPKs are expressed in almost all tissues [24]. However, some CDPK isoforms such as OsCDPK2 and OsCDPK11 have been found to be showing differential expression pattern under specific stimulus and stage of plant development [33]. Various enzymes and proteins have been identified as putative CDPK substrates utilizing *in vitro*  phosphorylation experiments. This includes a broad range of substrates, some of which include enzymes involved in carbon and nitrogen metabolism, ion channels and transporters, ethylene biosynthesis pathway and component of phospholipid metabolism [29]. Some of these substrates have been directly linked to the abiotic stress response-signaling pathway mediated by the CDPKs [34].

### CDPKs in Arabidopsis

Genome wide analysis of CDPKs in Arabidopsis has revealed the presence of 34 CDPKs. Many of these CDPKs are found to be showing differential pattern of expression under various developmental and abiotic stress conditions. Some of the CDPKs have been shown to be important in Abscisic acid (ABA) signaling which indicates their function in bringing about ABA-mediated abiotic stress response. AtCPK10 and AtCPK30 are known to activate an ABA inducible and stress related promoter [35]. HVA1 promoter (a barley stress promoter) used in this study was fused to green fluorescent protein (GFP) and the effect of constitutively active CDPKs was monitored in the transfected maize protoplasts. GFP expression indicating activation of this stress promoter even in the absence of stress signals provided evidence for the important role of CDPK activation in abiotic stress signaling cascade. Further, a deletion in the kinase domain in one of the two CDPKs, abolished GFP expression showing that the protein kinase domain is essential for stress promoter activation. *AtCPK10* and *AtCPK11* reported to be induced under drought and salt stress conditions indicating their possible role in osmotic stress signaling [36]. ABA is a stress specific phytohormone that is formed when plants encounter various environmental stress conditions and mediates signaling pathway, which helps generate adaptive responses to encounter such adverse conditions. Significant evidence has accumulated which shows the involvement of multiple CDPKs as positive regulators of ABA mediated signal transduction pathway under abiotic stress conditions. AtCPK32 regulates ABA mediated seed germination [37] while AtCPK3 and AtCPK6 were reported to be controlling ABA mediated closure of stomata [38]. *CPK3*  and *CPK6*  double mutant, *cpk3ckp6*  in Arabidopsis, was found to be impaired in activity of S (slow)-type anion channel in guard cells, which results only in partial closure of stomata in response to ABA [38]. The partial closure was due to partly overlapping functions of S-type and R-type of anion channels in the guard cell where R or rapid ion channel functions in a Ca^2+^-independent fashion.

ABF4, which is a transcriptional regulator of ABA responsive gene was phosphorylated at Ser^110^ by AtCPK32, AtCPK10 and AtCPK30 (CDPK subgroup III). ABF4 is a basic leucine zipper (bZIP) type transcription factor that interacts with the kinase domain of AtCPK32, and this domain although necessary for interaction with ABF4, is not sufficient alone since domains as well as the EF-hand domains are important for complete interaction [27]. Using deletion constructs and yeast two-hybrid studies, a conserved domain within the ABF4, namely, C2-C3 domain was determined to be the region responsible for interaction with AtCPK32. This again points towards an example where a CDPK might bring about the abiotic stress response by positively modulating its substrate (in this case a transcription factor). Overexpression of *AtCPK32*  confers hypersensitive response to ABA and salt stress conditions. Also, the expression of certain ABA responsive genes such as *RAB18*, *RD29A* and *RD29B* was up regulated in the overexpression lines, indicating the function of AtCPK32 as a positive regulator of ABA mediated stress-signaling pathway. Two more CDPKs from Arabidopsis, that include, CPK4 and CPK11, have also been shown to be positively regulating ABA signal transduction pathway as concluded from both mutant as well as overexpression studies [38]. *cpk4*  and *cpk11*  loss of function mutants displayed an ABA insensitive phenotype with respect to seed germination, seedling growth and root growth. Also, these mutants showed reduced sensitivity to ABA in terms of stomatal closure and had bigger stomatal aperture upon ABA treatment in comparison to the wild type. An opposite phenotype could be seen in the overexpression lines that showed increased sensitivity to ABA in terms of stomatal closure, seed germination, seedling and root growth. A similar contrasting pattern could be observed upon performing real time PCR to determine the expression of various ABA responsive genes in both mutant as well as the overexpression lines. Expression of several ABA responsive genes such as ABF1, ABF2, ABF4, ABA insensitive 4 (ABI4), and ABI5 was significantly downregulated in the mutant lines while considerable upregulation could be seen for these genes in the overexpression transgenic lines. *In gel*  phosphorylation assays demonstrated ABF1 and ABF4 to be phosphorylated by immunoprecipitated CPK4 and CPK11, which was reduced to a great extent in the *cpk4*  and *cpk11*  mutant lines. The molecular genetic evidence combined with biochemical approach employed in this study lends strong support to the role of CPK4 and CPK11 in ABA mediated signal transduction. As observed by the authors, AtCPK4 and AtCPK11 are localized in both cytoplasm and the nucleus, they could be participating in initial or early as well as delayed phase of stress response. Sensing of calcium fluxes brought about by stress stimuli, and phosphorylation of targets already present in the cell controlling responses such as stomatal closure could form an early response while phosphorylation of transcription factors at the nucleus affecting gene expression would form a later or delayed part of the response. In contrast to all these studies which show the role of CDPKs as positive regulators of stress signaling mediated by ABA, one of the CDPK gene, namely, *AtCPK23* is believed to be serving as a negative regulator of abiotic stress signaling as observed from the *cpk23*  mutant, which has improved tolerance to drought and salt stress [39]. However, whether this negative regulation of abiotic stress response pathway is *via*  negative control of *AtCPK23*  over ABA signaling pathway is not known.

Besides their role in ABA mediated signal transduction under stress conditions, the CDPKs could also function to relay ABA independent stress signaling as observed for AtCPK11 interacting with a zinc finger protein, namely, AtDi19-1 that was identified in a two hybrid screen. *AtDi19-1*  (drought induced) belongs to a novel stress regulated gene family (*AtDi19*) and encodes a zinc finger protein with nuclear localization signal/nuclear export signal (NLS/NES) sequences [40]. AtDi19 gene family members are believed to be participating in an ABA independent pathway during drought, salt and light signaling pathways as none of the members were induced upon ABA treatment. NLS/NES domain of AtDi19-1 is sufficient for both interactions with AtCPK11 as well as phosphorylation by AtCIPK11. Both NLS/NES and the C2H2 zinc finger domains are present in the central region of AtDi19. The NLS/NES domain could be important for shuttling both proteins between the nucleus and the cytoplasm during stress signaling. 

Control over calcium levels in the cell forms an important means of regulation of calcium mediated signal transduction pathways, and a report on AtCPK1 shows how calcium sensing CDPK could possibly play an important role in controlling calcium ions during signaling cascades [41]. CPK1 isoform in Arabidopsis was found to be inhibiting type IIB Ca^2+^-ATPase channel, which is a calmodulin-stimulated calcium pump (ACA2) (involved in calcium efflux from cytoplasm) present in the endoplasmic reticulum by phosphorylation at Ser^45^. This inhibition not only blocks the basal activity of this channel but also prevents its activation by calmodulin [41]. Here, crosstalk between two opposing pathways (by CaM and CDPK) was perhaps regulating the calcium levels and oscillations in the cell that might be crucial in regulating some specific responses to environmental stress stimuli.

Sub-cellular targeting of different CDPKs has provided insight into the possible functions specific to different isoforms of CDPK. CDPKs were localized at multiple cellular sites as demonstrated in different studies. GFP fusion constructs of nine AtCPK isoforms targeted in Arabidopsis roots has shown AtCPK3 and AtCPK4 to be having both cytoplasmic and nuclear distribution while AtCPK1, 7, 8, 9, 16, 21, and 28 are found to be associated with the membrane [42]. Of these, AtCPK1 was localized to the peroxisome where it was believed to be perhaps involved in oxidative stress and lipid metabolism. Besides AtCPK1, the other membrane anchored CDPK isoforms were found to be present in the plasma membrane. AtCPK2 that has high similarity to AtCPK1, was also shown to be localized in the endoplasmic reticulum (ER), and is believed to be having a similar potential substrate as AtCPK1, namely, ACA2 which is also localized at ER [43].

### CDPKs in Rice

A large multigene family has also been identified in rice comprising of 31 CDPK genes in rice genome annotation and transcriptomic analysis [44, 45]. Studies aiming to uncover the expression profile of these multiple genes at both global as well as single gene level highlight the relevance of CDPKs in abiotic stress and development. 

RT-PCR (Reverse transcriptase-PCR) and RNA gel blot studies [44] have indicated the presence of 17 OsCPK transcripts in response to cold, drought, salt and heat stress. These include *OsCPK1*, *OsCPK4*, *OsCPK*  *6*, *OsCPK7*, *OsCPK8*, *OsCPK9*, *OsCPK10*, *OsCPK 13*, *OsCPK14*, *OsCPK15*, *OsCPK16*, *OsCPK17*, *OsCPK19*, *OsCPK23*, *OsCPK24*, *OsCPK25*  and *OsCPK29*. Of these, four genes show differential response to these stresses. *OsCPK17*  was down regulated by cold, drought and salt stress. *OsCPK6*  was up regulated by drought and *OsCPK25*  was showing up regulation in response to heat stress. *OsCPK13*  (encoding OsCDPK7) was up regulated by salt, drought and cold stress. Microarray based expression studies of rice seedling grown under cold, drought, and salinity stress for 3 hours time respectively has revealed the induction of six CDPK genes (*OsCPK4, 10, 12, 13, 15, 21*) and down regulation of one gene *OsCPK1*  [45]. *OsCPK13*  was induced under all stress conditions*. OsCPK4*  was induced under cold stress while the rest of the CDPK genes, *OsCPK 10, 12, 15*  and *21*  were up regulated specifically under desiccation stress. Least up regulation was observed under salt stress where none of these genes showed more than 2 fold change in expression. In fact, *OsCPK1*  transcript levels were found to be decreasing by 2 folds under salinity stress. The lack of correlation in the expression profile for some of the rice CPKs such as *OsCPK6*  studied from RNA gel blots and microarray could possibly be due to difference in the rice variety and experimental conditions employed in these studies. Additional support for the involvement of CDPKs under abiotic stress conditions has come from the analysis of cis-elements of the CDPK genes [44]. Of the different elements studied, the rice CDPK genes were found to be having one or more number of such different 1kb elements upstream of the gene. Most of the rice CDPK genes were found to be having an EBOX cis-element (EBOXBNNAPA), which is having an ABA responsive element (ABRE) motif characterized by the sequence CANNTG. Presence of such cis-elements upstream of the CDPK genes points towards the ability of such sequences to bind with stress specific transcription factors such as dehydration responsive element/C-repeat (DREBs/CBFs) that can then control the expression of stress responsive CDPK genes under various dehydration stress conditions such as low temperature, salt and drought. 

Previous studies at the level of single genes have conclusively shown the direct role played by some of the CDPKs in abiotic stress signaling cascades. OsCDPK13 and OsCDPK7 are rice CDPKs for which expression and functional analyses has been carried out in detail in different studies [46, 47]. OsCDPK13 gene is induced by cold and gibberellin in sheath as well as in callus as determined by northern blot analyses [46]. However, the mRNA levels for this gene were suppressed by drought and salt stress along with brassinolide and ABA treatment. Consistent with this observation, a similar pattern of CDPK13 protein expression was seen from immunoblot experiments. Additional studies have implicated OsCDPK13 (encoded by *OsCPK7*) as a mediator of both gibberellic acid (GA) and cold signaling [48]. Both GA and cold stress are known to be inducing changes in cytosolic Ca^2+^ levels [1], and thus said to be involved in calcium-mediated signaling. Some other proteins such as calreticulin, are also perhaps involved in the cold tolerance pathway mediated by OsCDPK13 and calreticulin interacting protein1 (CRTintP1). This was concluded from 2D-polyacrylamide gel electrophoresis (PAGE) profiles of transgenic lines overexpressing OsCDPK13 and CRTintP1, both of which, showed higher levels of calreticulin [49]. Also, cold tolerant varieties had higher levels of both of these proteins. Proteomic analysis of these overexpression lines revealed the upregulation of fructokinase, cytoplasmic malate dehydrogenase and tubulin. Such proteins might have a protective role in cold stress response by repairing damage caused under such conditions. Subcellular localization study using immuno-localization experiments showed OsCDPK13 to be a cytosolic protein [46]. Another CDPK (encoded by *OsCDPK14*) was also found to be localized in the cytoplasm and is believed to be involved in mediating calcium signaling in the cytosol [50].

Overexpression of OsCDPK7 confers the rice plants with enhanced tolerance to both cold as well as salt stress [51]. In the same study, both *in situ*  hybridization experiments to detect mRNA levels and immuno-localization study to detect protein levels showed that it mainly expressed in vascular tissues of crowns and roots, vascular bundles and central cylinder, respectively. These are the tissues where water stress occurs most strongly, and overexpression of OsCDPK7 is seen to be causing higher transcript accumulation of not only OsCDPK7, but also increased accumulation of *rab16A*, which is a putative target gene of OsCDPK7. OsCDPK7 has been proposed to be serving as a branch point of cold and salt/drought signaling pathway acting upstream of these target genes such as *rab16A*.

Most of the CDPKs also showed a tissue specific expression as determined from various studies in rice, Arabidopsis, and wheat. For example, in rice, expression of 19 CDPK genes was detected using northern blot and RT-PCR based studies in roots, stems, leaves and panicles [44]. A comprehensive expression profiling of 31 CDPK genes in rice employing microarray has been performed [45] in three vegetative stages, six stages of panicle and five stages of seed development. Following this, the genes have been grouped into eleven categories with genes showing maximum expression levels being placed in the first category. Twenty-three transcripts were found to be showing a differential pattern of expression in the reproductive development stages. *OsCPK23*  showed maximum up regulation of up to 87-folds in panicle stages and 1,724 folds in the seed development stages relative to its expression in mature leaf. Some genes like *OsCPK5*  and *16*  showed up regulation during panicle development and were down regulated at the seed stage. In contrast, *OsCPK8*  transcript levels were increased in the seed developmental stage and decreased in the panicle stages. Genes such as *OsCPK1, 4*, 13, 15, *19*, *20*, and *24* were specifically down regulated in late stages of seed development. *OsCPK24*  was found to be specifically upregulated in S1 stage of seed development, and has earlier been shown to be upregulated during wounding [44]. As proposed by the authors, early seed development involving pollen tube growth is known to be similar to wounding response or invasion by fungal hyphae, and that both elicit similar responses [52]. *OsCPK9*  had highest expression in the mature leaf with undetectable transcript levels in the panicle stages. Opposite to this, *OsCPK21*  was observed to be having almost no expression in the vegetative stage, but higher transcript level was observed in seed and panicle stages correlated with the presence of a cis-regulatory element motif required for endosperm expression in *OsCPK21*. All the genes that were placed in the eighth category expressed in the P6 stage of panicle development, these include *OsCPK6, 14, 22, 25*, *26*, and *29*  suggesting their expression in mature pollen. Also, *OsCPK25*  and *26*  had high similarity to a maize CDPK gene which also expressed in the late panicle stages, and whose inhibition by antisense mechanism resulted in impaired germination and pollen tube growth [53]. Thus, *OsCPK25*  and *26*  together with other genes of this category could be specifically involved in pollen maturation. Still others that include *OsCPK11*, *27*  and *31*  were found to be showing almost no expression, which could possibly be due to some other specific stimuli, or stage that may be responsible for inducing the expression of these genes. Also, expression pattern for some genes (*OsCPK2*, *14*, *21*, *23* and *29*) has been further confirmed by quantitative PCR, and was found to be showing a good correlation with microarray expression profile for these genes. *OsCPK2*  was detected at higher levels only at the P6 stage in the microarray analysis which was in agreement with RNA gel blot studies that also showed the presence of this transcript specifically at the panicle stage [44, 45].

Microarray based expression analysis of some CDPK genes under stress conditions has also been documented in RED (Rice Expression Database; http://red.dna.affrc.go.jp/RED/). Both *OsCPK*  *7*  and *15*  are not regulated by salt stress in the 7 days old leaf stage as indicated by their relative expression values close to 1. However, at 24 days old leaf stage, under salt stress conditions, the expression of *OsCPK7*  was seen to fall below 1 while that of *OsCPK15*  was increased by more than 1 [44]. This indicates that some isoforms of CDPKs are possibly devoted to abiotic stress tolerance in specific tissue or development stage.

All such detailed expression profiling of the CDPK gene transcripts showing their differential regulation underlines the functional role and importance of this large family of calcium sensing kinases in both abiotic stress and different development stages in rice, which is an important model crop plant, and provides an avenue to carry out further functional characterization of these genes using other molecular tools and techniques.

### CDPKs in Wheat and other Plants

In addition to rice, genome wide analysis and expression studies of CDPKs has been performed in wheat where 20 CDPK genes have been identified based on hidden markov model (HMM) generated using rice CDPK proteins followed by full length cDNA isolation for some of these genes [54]. Based on such analysis, detailed features of the 20 CDPK genes identified has been elucidated with respect to details such as the number of EF-hands, protein length, subgroup classification and N-terminal amino acids along with information regarding the presence of myristoylation motif and subcellular localization. Like Arabidopsis and rice, the wheat CDPKs also showed a tissue specific expression pattern, and 13 of the CDPKs studied exhibited variable pattern of expression in the leaf, root, stem, young spikes and immature seeds as tested by semi quantitative RT-PCR. *TaCPK1*, *2*, *5*, and *16*  were detected in all the tissues. *TaCPK3*, *6*, *9*, *12*, *14*, and *15*  were found to be expressing mainly in the root, stem, leaf and young spikes. *TaCPK10*  was expressed significantly in all the tested tissues except stem [54]. A more limited expression pattern was observed for *TaCPK8*  (predominant expression in leaves and immature seeds) and *TaCPK13*  (mainly expressed only in young spikes). Such specific expression pattern in defined developmental stages point toward the role of CDPKs in plant physiology and development, besides their crucial role in abiotic stress response. An elaborate study to determine the expression of the wheat CDPK genes under abiotic and biotic stress was also undertaken. Seedling tissues exposed to abiotic stresses such as cold, drought, salt, hydrogen peroxide (oxidative stress), and hormones such as ABA and GA were observed for specific expression of the identified CDPK genes. Multiple CDPKs were found to be expressing under a given treatment and different stress treatments were also seen to be inducing the expression of some common CDPKs, implying the possible cross talk or common point, which might exist between such different stress signaling pathways. Maximum numbers of the identified genes were responding to hydrogen peroxide as an abiotic stress as determined by the differential expression of *TaCPK1*, *2*, *5*, *7*, *9*, *10*, *12,* and *18* using RT-PCR. Cold stress was seen to be affecting the expression of seven *TaCPK* genes, namely, *TaCPK3*, *4*, *5*, *6*, *7*, *12*, and *15*. Five genes, *TaCPK 4, 9, 10, 18, 19*  responded to salt stress while only four genes, *TaCPK1*, *6*, *9*, and *18* were shown to be affected by drought [54]. ABA commonly associated with stress responses was seen to be causing the induction of *TaCPK3*, *4*, *5*, *6*, *7*, *9*  and *10*. Expression of one of the CDPKs, *TaCPK4*  was common to all the tested hormones and abiotic stress conditions except drought. In fact, *TaCPK4*  expression was also observed under the biotic stress condition tested that involved infection with the fungal pathogen *Blumeria*  *graminis*  *tritici*  (causal agent of powdery mildew). TaCPK2 and OsCPK13 share high identity at the level of protein sequence and both of these were detected in leaf, stem and root tissues. However, comparison of the expression of *TaCPK2*  [54] with *OsCPK13*  (a previously well studied CDPK gene in rice identified as a putative *TaCPK2*  ortholog) revealed that both respond to pathogen challenge in their respective host, but unlike *OsCPK13*, *TaCPK2*  appears to have lost its ability to respond to cold, drought and salt stress conditions.

To correlate the *in silico*  structural features of the wheat CDPKs like the predicted myristoylation motif with their respective *in vivo*  function such as membrane association, subcellular localization studies utilizing onion epidermal cells were performed [54]. Out of the 12 TaCDPKs tested for their localization, seven TaCDPK-GFP constructs were found to be giving the fluorescence signals. TaCPK2 and 5 that had previously been identified to be containing a myristoylation motif were found to be membrane associated. TaCPK3 and 15, which did not harbor such myristoylation motif were also observed to be localizing to plasma membrane [54]. This showed that other membrane association motifs might be present that were not detected by the *in silico*  methodology. Both TaCPK3 and TaCPK15 were determined to be consisting of a bipartite NLS or nuclear localization in their junction domain, which according to the authors indicated the possibility of migration of these CDPKs from the plasma membrane to the nucleus during signaling [54]. Similarly, TaCPK1, 4, and 9 that were not found to be having any NLS sequence or myristoylation motif [54] were seen to be both plasma membrane as well as nuclear localized, indicating the presence of uncharacterized motifs which are responsible for the observed localization pattern.

Various studies in other plant species have also established the role of CDPKs under environmental stress conditions. *PaCDPK1*  from Orchid (*Phalaenopsis amabilis*) was induced by cold, wounding and pathogen attack as shown by promoter analysis of this gene utilizing β-glucuronidase (GUS) expression study as well as RNA blot experiments to detect the transcript levels. Northern blot hybridization showed high levels in labellum and peloric flowers [55]. *VfCPK1*  from *Vicia faba*  or broad bean showed increased accumulation upon drought stress and ABA treatment. Analysis in different parts of this plant showed *VfCPK1*  expression to be particularly high in leaves especially the epidermal peels [56]. NtCDPK1 from *Nicotiana tabacum*  is detected largely in rapidly proliferating tissues such as roots, stems, and flowers. It was also induced by salt, wounding, calcium, ABA, GA, cytokinin, jasmonate, fungal elicitors and chitosan reflecting its possible role in a wide variety of stress and hormone signaling pathways [30]. Immuno-precipitation studies found NtCDPK1 to be associated with the membrane fractions. *NtCPK4*  is another CDPK from tobacco that has an important function in development as well as abiotic stress response as concluded from the transcript levels for this CDPK that showed a time and space dependent expression in the vegetative and reproductive tissue [57]. Higher expression was detected in zone of cell division and vascular bundle. A varied expression was seen in different parts of the flower where large amount was detected in the tapetum and anther wall. During the pollen development, level of *NtCPK4*  gradually declined. Similarly during ovule development, it was present in higher level in placenta, ovule primordia and ovule. After fertilization, it was seen to be accumulating in the embryo where a peak in the expression was detected at the pro-embryo stage. Increased *NtCPK4*  mRNA accumulation was also seen after salt and GA treatment. *LeCDPK1*  from tomato was observed to be induced upon mechanical wounding [58], and was also shown to be conferring the plants with salt tolerance [59]. This was a type of cross-tolerance where resistance to one type of stress provides an additional resistance to another form of stress. *LeCDPK1*  transcripts were also seen to be increasing upon both salt and ABA treatment. Jasmonic acid (JA) treatment enhanced the expression of *LeCDPK1*  mRNA which indicates that perhaps during wounding, it was JA signaling pathway that upregulates *LeCDPK1*  expression whereas during abiotic stress such as salinity, it was ABA that activated its expression. *Mesembryanthemum crystallinum*  calcium dependent protein kinase 1 (*McCPK1*) transcripts were induced in response to salt and dehydration stress [60]. McCPK1 also exhibited an interesting localization pattern where in response to salt and dehydration stress, it was seen to be moving from the plasma membrane to the nucleus. McCPK1 interacts with McCAP1 as determined from yeast two-hybrid studies, which was possibly a cytoskeletal protein. Another CDPK from mungbean, *VrCDPK*  was expressing under mechanical stress [61] while *MsCPK3*  (in alfalfa) was induced under heat stress [62]. 

Several potential CDPK substrates have been identified, some of which provide a clue towards the downstream targets of CDPKs in abiotic stress signaling. Many ion transporters and channels have been found to be serving as substrates for CDPKs. A 59 KDa CDPK from *Vicia faba*  guard cell has been reported to be phosphorylating and inhibiting the K^+^-inward channel, KAT1 [34]. This kind of targeting of the potassium channel might point towards the role of CDPKs in mediating stomatal closure during ABA signaling that occurs during stress conditions. Similarly, another CDPK from the guard cells of *Vicia faba*  has been shown to be activating an influx chloride channel on the vacuole. However, in the latter case it was still not clear if the CDPK acts directly to activate the channel or if it first phosphorylates an intermediate regulatory protein, which then activated the channel. Here, ABA biosynthesis was up regulated under stress conditions such as drought and has been shown to be involved in mediating signal transduction pathway for stomatal closure [34]. Calcium plays an important role in the direct activation of CDPK kinase activity as demonstrated *in vitro*  in a few studies [30, 57].

## CALCINEURIN B-LIKE INTERACTING PROTEIN KINASE (CIPK) AND ABIOTIC STRESS 

CIPKs are a class of novel serine-threonine protein kinases in plants involved specifically in interacting with calcium sensing protein calcineurin B-like protein (CBL) and transducing the various abiotic stresses, nutrient and hormonal signals further downstream in the signaling pathway [9, 12, 22, 63, 64]. Recently, significant evidence has come which suggests their role in developmental processes [65, 66]. CIPKs belong to the SnRK3 subgroup of protein kinases and show similarity at the structural level to SNF1 (sucrose non-fermenting) kinase from yeast and AMPK (AMP activated protein kinases) from animals. CIPKs have a catalytic N-terminal kinase domain and a regulatory C-terminal domain. Another domain, which was highly conserved, is the NAF/FISL motif, which has a stretch of 24 hydrophobic amino acid residues, and is responsible for mediating interaction with CBLs. Also, the NAF domain serves to inhibit the kinase activity of CIPKs, which is relieved by the binding of CBLs to this domain [9, 12, 22, 63]. Since CIPKs do not have any localization motif of their own, it is the myristoylation/palmitoylation motifs present within CBLs that help anchor CIPKs to the membranes [67]. The kinase activity of CIPKs was confirmed using commonly employed substrates for kinase assay such as myelin basic protein (MBP) and histone IIIS that were found to be phosphorylated by OsCK1 which is a stress responsive CIPK member from rice [68]. Besides, many other physiological substrates for CIPKs such as potassium channel (AKT1) and Na^+^/H^+^-antiporter (SOS1) were phosphorylated by CIPKs confirming the *in vivo*  kinase activity of CIPKs [69, 70].

### CIPKs in Arabidopsis

Genome analysis in Arabidopsis has revealed the presence of 26 CIPKs in Arabidopsis [71]. RT-PCR based cDNA cloning of all the AtCIPKs confirmed their expression and also validated the theoretically predicted gene structure for some while leading to the correction for others. Since CIPKs do not sense calcium directly, and require CBLs as calcium sensors to relieve the inhibition of their kinase activity, protein interaction analysis using yeast two hybrid approach has been utilized to identify potential CBL-CIPK interacting partners to gain a clue about interactions which could actually be taking place *in vivo*  in response to abiotic stress. Yeast two-hybrid analysis to study the interaction of Arabidopsis CBL1 and CBL9 with all the CIPKs have shown that CBLs that are closely related in sequence may not necessarily interacts with similar subset of CIPKs [71]. For instance, only four AtCIPKs (AtCIPK1, 8, 18, 23 and 24) exhibited strong interaction with both AtCBL1 and 9. Others such as AtCIPK21 interacted more significantly or strongly with AtCBL9 while AtCIPK7 and 17 preferentially interacted with AtCBL1. Some of the CIPKs such as AtCIPK5, 11, 12 and 23 interacted weakly with AtCBL1. Of these, AtCIPK5, 11 and 12 exhibited a weak interaction even with AtCBL9 along with other weakly interacting partners, which include AtCIPK6, 10 and 16 [71]. Another study [72] has found AtCBL3 to be interacting specifically with AtCIPK11 in a calcium dependent manner. This shows that each CIPK could be interacting with a specific subset of CBLs at varying strengths, and that phylogenetic relationship was not indicative of CBL-CIPK interacting partners as concluded from the observed interaction of evolutionarily separated intron harboring (AtCIPK1, AtCIPK8, AtCIPK17 and AtCIPK24) and intron free (AtCIPK7 and AtCIPK18) with AtCBL1.

CIPKs are found to be differentially regulated by a variety of conditions as demonstrated by their expression pattern under cold, drought, salt, ABA, low K^+^ ion conditions and various developmental stages [21, 65, 73, 74]. Studies based on mutant analysis under different stress conditions involving stress marker gene expression pattern, seed germination assays on stress media combined with biochemical approach have clearly implicated different CBL-CIPK partners to be involved in sensing specific or multiple stress conditions by modulating different downstream targets such as potassium channels (AKT1), sodium efflux pumps (Na^+^/H^+^-antiporter) and other proteins with as of yet unknown function such as ECT1 [69,70,75]. Recently, the role of CIPK in development has been clearly defined from studies based on specific CIPK members such as *AtCIPK6*  from Arabidopsis [65].

In CIPK gene family in Arabidopsis, *CIPK3*  transcript was strongly inducible by cold, drought, salinity, wounding and ABA [73]. One of the important report by Hu *et al*. 2008 [64], showed the involvement of CIPK8 in early nitrate signaling. CIPK8 expression profiling in Arabidopsis plants by quantitative PCR showed that CIPK8 was induced very rapidly by NO^-3^. The other CIPKs which were responsive to abiotic stress include, *CIPK9*  (responsive to low potassium levels) [21], *CIPK11 /PKS5*  which is responsive to ABA, drought and salinity [76], and *CIPK21*  that is weakly inducible by ABA and abiotic stresses (Pandey *et al.*, unpublished). Beside *CIPK9*, which was strongly up regulated under potassium deprivation stress condition [21], the transcript of another member of this family, *CIPK23*, was also slightly up regulated under potassium deficient condition [77]. Moreover, some of these CIPKs were also regulated spatially and temporally and at different developmental stages of plants [9, 12].

Numerous studies at the level of single genes have elucidated the role of CIPK in abiotic stress conditions. Both CBL1 as well as CBL9 were found to be interacting with CIPK1 as studied by yeast two-hybrid method and this interaction was seen to be targeting CIPK1 to the plasma membrane which was determined from GFP localization studies in *Nicotiana benthamiana*  protoplasts [74]. Further, *cipk1*  mutants like *cbl9*  mutants exhibited hypersensitive response to ABA while *cbl1*  mutant was not affected in ABA response. This showed that CIPK1 upon interaction with CBL9 serves as the negative regulator of ABA response under osmotic stress conditions. Such experimental data indicated the presence of a plausible pathway where alternate complex formation of CIPK1 with either CBL1 or CBL9 decides the activation of an ABA dependent or ABA independent pathway under abiotic stress.

CIPK3 has been shown to be interacting with CBL9 [78] was earlier shown to be regulating ABA and cold signaling pathway in Arabidopsis [73]. *cipk3*  mutant seed germination was found to be inhibited more by ABA and salt than the wild type plants which suggested its role as a negative regulator of ABA response [78]. Like CIPK1, CIPK3 was also shown to be interacting with CBL9 in bringing about this response as concluded from ABA hypersensitive phenotype observed in *cbl9*  mutants which was rescued by the overexpression of constitutively active CIPK3 but not by that of the wild type CIPK3 indicating that CIPK3 functions downstream of CBL9 which serves to activate CIPK3 in response to ABA during seed germination [78]. Expression analysis by RNA gel blot of *CIPK3*  under ABA and other abiotic stress treatment indicated that it was strongly induced under ABA, salt, drought, cold as well as wounding. GUS expression analysis and RT-PCR of different developmental stages showed that the expression of *CIPK3*  was highest in the seedling stage in contrast to the other adult stages such as mature leaf, stem, root, flower and silique, which had lower expression of this gene. This expression pattern of CIPK3 reflects its role and importance under stress conditions in the seedling stage as determined from mutant studies, and might also indicate its as of yet unknown function in development. To test the effect of *CIPK3*  mutation at the downstream level of stress signaling pathway, several of the stress marker genes were studied. On comparison between the *cipk3*  mutant and wild type plants, it was concluded that for some of the genes such as *KIN1*, *RD29A/B*  under cold and salt treatment, the early period of induction was being controlled by *CIPK3*. *CIPK3*  perhaps regulates an ABA independent salt stress pathway since no effect on stress gene expression was observed in case of *cipk3*  mutant plants that were given drought stress, and because salt and drought have a common ABA dependent pathway, so it can be concluded that *CIPK3*  brings about salt stress response in a manner which is independent of ABA. More importantly, CIPK3 is believed to be acting as a cross talk node for ABA, salt and cold stress signaling since in *cipk3*  mutants, expression pattern of the stress marker genes was simultaneously altered in all these conditions. Interestingly as noted by the authors, CIPK3 is possibly a cross talk node for cold and ABA signal transduction that were previously thought to be independent of each other. Based on this study, it can be postulated that ABA together with cold has a synergistic effect by inducing greater changes in calcium levels which would then activate higher levels of CIPK3 node and bring about cold induced gene expression which would otherwise be impaired or reduced in ABA deficient mutants.

Another report also establishes the function of CIPK15/PKS3, which interacts with CBL1 as a negative regulator of ABA signaling pathway [79]. In this study, silencing of both *CBL1*  and *CIPK15*  resulted in ABA hypersensitive phenotype. Also, CIPK15 was found to be interacting with PP2C phosphatases, ABI1 and ABI2, which were speculated to be interacting with other downstream targets or proteins modulating ABA regulated gene expression. Thus, CIPKs together with CBLs could be having an opposite effect on ABA signaling as compared to CDPKs, which promote or serve as a positive modulator of ABA signaling.

Potassium deficiency is a form of nutrient stress in which involvement of CIPK9 has been elucidated [21]. *CIPK9*  expression was studied under various abiotic stress conditions using semi-quantitative RT-PCR, and it was found to be induced under osmotic stress, salt, cold, wounding and low potassium conditions (low-K^+^ stress was provided by eliminating KCl from the MS medium). Similar analyses for *CIPK9*  expression in different tissues were conducted using RT-PCR and GUS expression analysis. Anthers, stigma, petals, sepals and siliques in particular, showed a strong expression of this gene. Increase in *CIPK9*  transcript accumulation was seen till after 5 days of low potassium stress. Such a strong induction of a CIPK gene under low potassium stress was perhaps unique to *CIPK9*, which might be specific to this form of nutrient stress. This was supported by the fact that T-DNA mutant lines of *cipk9*  were specifically altered in their response to low potassium stress and exhibited a hypersensitive response under low-K^+^ conditions.

Other studies have also shown CBL-CIPK members to be involved in low potassium signaling. For instance, CIPK23 has been implicated in potassium signaling where it was proposed to be acting by interacting with upstream calcium sensors such as CBL1/CBL9 as shown by yeast two-hybrid studies that was confirmed using bimolecular fluorescence complementation assays (BiFC). Such interaction brings about phosphorylation of K^+^ transporter channel, AKT1 [69, 80, 81]. However, CIPK9 has a different mode of action in this respect since no change in K^+^-uptake was seen in *cipk9*  mutants, and it can thus be said that CIPK9 does not directly interact with the K^+^-transporters involved in potassium uptake. An effort has been made to determine the interacting partners of various CIPKs using yeast two-hybrid studies. In comprehensive yeast two-hybrid study, two more CIPKs, CIPK6 and CIPK16 were also found to be interacting with AKT1 in addition to CIPK23. Similarly, two more CBLs, CBL2 and 3 in addition to CBL1 and CBL9 already reported in earlier studies [80,81], were found to interacting with all three CIPKs, namely, CIPK6, CIPK16 and CIPK23 [69].

Like CIPK1, CIPK24 or salt overly sensitive2 (SOS2) is another calcium signaling kinase that mediates salt signaling pathway by associating with CBL10 in the tonoplast [82] or CBL4 in the plasma membrane [83]. The regulation of ion homeostasis by CBL10 follows a unique mechanism as revealed by the *cbl10*  mutants that show lower Na^+^ ions under both normal as well as high salt conditions unlike other salt sensitive mutants [82]. Yeast two-hybrid experiments identified CIPK24 as a strong candidate for interacting with CBL10 which was further confirmed utilizing GST fusion constructs of CIPK24 that co purified CBL10 in the pull down assay. BiFC proved the localization of CBL10-CIPK24 at the vacuolar membrane. Such low Na^+^ ion content may be due to sequestration of these excess Na^+^ ions in the vacuole, a mechanism most suitable for shoot tissue where CBL10 is primarily expressed. In contrast, CBL4, which localizes to the cell membrane, and was strongly expressed in roots, might be mediating the export of excess sodium ions outside into the soil *via*  interaction with CIPK24 to activate Na^+^/H^+^-antiporter [83]. CIPKs have been found to be playing a critical role in cold, drought, salinity and ABA responses, and target proteins for some of the CBL-CIPK complexes have been identified. The Na^+^/H^+^-antiporter or SOS1 is a well-established target identified for the SOS3 (CBL4) and SOS2 (CIPK24) complex that is activated, and functions under salt stress [20, 84]. Response of *sos1, sos2* and *sos3*  mutants under salinity stress indicated the involvement of these three proteins in a common signaling pathway regulating salt toxicity. s*os3*  mutant did not show up regulation of SOS1 mRNA under salt stress in either root or shoot whereas *sos2*  mutant exhibited this loss in up regulation of SOS1 in roots but not in shoots which can be explained by the fact that CBL4 that is plasma membrane localized functions mainly in the salt tolerance pathway active in the root tissue [85]. *sos1*  mutant displayed greater sensitivity towards Na^+^ and Li^+^ stress in comparison with *sos2*  and *sos3*  plants [86]. SOS1 was identified to be a 127 kDa Na^+^/H^+^-antiporter that was related to plasma membrane Na^+^/H^+^-antiporters found in bacterial species such as *E. coli* and *Pseudomonas aeruginosa*. SOS2 and SOS3 are believed to be activating the Na^+^/H^+^-antiporter in response to NaCl and subsequent rise in calcium levels caused by the stress. SOS1 functions to pump sodium ions out of the cytosol into the environment, and hence protects the cell from salt stress. A rice homolog of SOS1, namely, OsSOS1 demonstrated to be phosphorylated by AtSOS2 and AtSOS3 complex [70]. Yeast two-hybrid approach has identified yet another transporter H^+^-ATPase which is present in the vacuole, to be interacting with SOS2 [87], and regulating plant responses to salt stress since such interaction was found to be enhanced under salt stress conditions.

CIPK11 interacting with CBL2 was found to be regulating a plasma membrane H^+^-ATPase referred to as AHA2. In the study (by Fuglsang *et al*. 2007) [76] using reverse genetics approach, it was found that AHA2 was negatively regulated by PKS5 or CIPK11, which phosphorylated the pump at Ser^931^. This prevented the activation of AHA2 by 14-3-3 proteins that binds to phosphorylated Thr^937 ^of this proton pump. In *pks5*  mutants utilized for this study, the PM (plasma membrane) H^+^-ATPase pump was de-repressed in mutant, and enhanced the survival of plants under high pH. This occurs primarily due to the ability of AHA2 to pump protons outside into the extracellular space that helps in acidification of the local rhizosphere. Under normal conditions, repression by CIPK11 might be useful for membrane depolarization and cytoplasm acidification in response to external stimuli [76]. 

A more recently identified class of novel CIPK interacting proteins are ECT1 and ECT2 which are named so due to the presence of a conserved 180 amino acids long C-terminal domain. This class of protein was found in eukaryotic organisms such as monocot plants, yeast and humans, and around 11 such ECT proteins were found in the Arabidopsis genome. These two proteins have been reported as the target for CIPK1 [75], based on yeast two-hybrid screening and pull down assays. Using deletion constructs, it was found that the C-terminal domain of CIPK1 alone was sufficient for interaction with ECT proteins. Similarly, the C-terminal conserved residues in ECT1 and ECT2 were enough for this interaction. Also, it was found that only ECT1 and ECT2 specifically interact with CIPK1 since other tested ECTs (ECT3, ECT6, ECT10 and ECT11) were not found to be showing interaction with CIPK1. In addition, only ECT11 was interacting with CIPK3. Further, it was found that CBL1 and CIPK1 co-expressed with ECT1 are localized into the nucleus. A nuclear localization signal was subsequently found in the C-terminal domain (rich in positively charged amino acids) of ECT proteins. Detail molecular analysis of these proteins by genetic and molecular approach will shed more light in their role in calcium mediated abiotic stress signaling pathway, which need more experimental input.

Very recently, Tripathi *et al*. [65] have identified the role of Arabidopsis CIPK6 in plant growth and development. A mutation in *AtCIPK6*  significantly reduced shoot-to-root and root basipetal auxin transport, in conjunction with reduced expression of a number of genes involved in auxin transport and abiotic stress response. Also, the plants exhibited developmental defects such as fused cotyledons, swollen hypocotyls and compromised lateral root formation The Arabidopsis mutant was more sensitive to salt stress as compared to wild type, and overexpression of a constitutively active mutant of CIPK6 promoted salt tolerance in transgenic tobacco.

In an interesting study by Hu *et al*. [64] the possible function of another CIPK family member, AtCIPK8 was determined, which was seen to be up regulated by nitrate mineral nutrient, and expression of nitrate-regulated gene transcripts of several nitrate transporters in *cipk8*  mutant plant were affected. Induction of *CIPK8*  transcripts by nitrate was predicted to be positively regulating nitrate signaling. This study provided the first evidence for the involvement of a CIPK in nutrient sensing besides their function in abiotic stress and potassium deficiency.

### CIPKs in Rice

Comparison of Arabidopsis CIPKs with ESTs of two rice subspecies (*Oryza sativa* subspecies *japonica*  and *Oryza sativa* subspecies *indica*) led to the identification of 30 CIPKs in both these rice varieties. In addition to the homology, other structural features such as the occurrence of NAF domain were taken into account for uncovering CIPK genes in rice [71]. *OsCK1*  isolated by a differential cDNA screening approach was strongly induced by cold, light, salt, sugar and cytokinins [68]. Of the various tissues tested, significant expression could be detected only in shoots. The expression profiling of *OsCIPK1-30*  under ABA, cold, drought, polyethyleneglycol (PEG) and salinity has shown most of them to be inducible by at least one type of these stress or stimuli. RNA gel blot as well as real time PCR and RT-PCR based experiments were conducted to detect the rise in *OsCIPK*  transcripts if any under these stress conditions [88]. It was found that of the 30 CIPKs, 27 *OsCIPK*s (except *OsCIPK13*, *14* and *27*) were detected either in the RNA blot /PCR or both. Out of 27 *OsCIPK*s, 20 were induced under various stress stimuli. 15 genes (*OsCIPK1*, *2*, *5*, *9*, *11*, *12*, *15*, *17*, *20*, *21*, *22*, *23*, *24*, *29*, and *30*) responded to drought, 12 genes (*OsCIPK7*, *8*, *9*, *10*, *11*, *15*, *16*, *17*, *21*, *22*, *23*, *24*  and *29*) were salt inducible, 12 of these genes (*OsCIPK1*, *3*, *9*, *12*, *15*, *16*, *17*, *21*, *22*, *23*, *24*  and *29*) were induced by PEG, 16 of the *OsCIPK*s (*OsCIPK*  *1*, *2*, *3*, *5*, *7*, *9*, *11*, *12*, *15*, *16*, *17*, *20*, *22*, *24*, *29* and *30*) were ABA inducible, and only 3 genes (*OsCIPK1*, *3*  and *9*) showed response to cold. As the data indicates, some genes like *OsCIPK1, 3, 9, 15, 16, 17, 21, 22, 29* and *30* were induced by at least three of the tested stress conditions while others such as *OsCIPK3*  show significant induction under cold stress with small rise on ABA and PEG treatment [88]. In addition, most of the genes regulated by salt stress were also induced by drought while those *OsCIPK*  genes that were induced by either ABA or PEG, also showed response to both drought and salt stress. This observation as noted by the authors lends support to the concept of cross-talk or interaction between different abiotic stress signaling pathways where most of the genes mediating response to drought or salt also bring about the response to ABA which is a stress hormone. Since many of the abiotic stress conditions cause dehydration and osmotic stress, therefore, common CIPKs might be induced in that response. At the same time, many of the *OsCIPK*s that were induced by salt or drought do not respond to ABA [88]. This again hints toward the possible existence of ABA and ABA-independent pathway in plants for sensing the abiotic stress conditions.

For *OsCIPK03*, which shows prominent expression under cold stress conditions alone, transgenic lines of *japonica*  rice var Zhongua11 overexpressing this gene were generated and these plants were found to be exhibiting an improved tolerance to cold [88]. The transgenic lines were found to be having a better survival rate in comparison to the wild type rice plants. Similarly, overexpression of *OsCIPK12*  and *OsCIPK15*  rendered the transgenic rice plants with enhanced survival rates under drought and salt stress conditions respectively. Further, to examine the reason for such resistance that is brought about by these genes, the level of proline, which is an osmolyte, as well as that of soluble sugars was tested [88]. In both *OsCIPK03*  and *OsCIPK12*  overexpressing transgenic lines, higher accumulation of both these substances was found, which correlated with higher expression levels for two proline biosynthesis genes as well as for two proline transporter genes [88]. Such improvement in tolerance toward the abiotic stresses achieved by higher overexpression of these CIPK genes establishes the role and importance of the CBL-CIPK signaling pathway as a tool for creating transgenic plants resistant to environmental stresses.

A recent study has shown the overlapping role of a rice *CIPK*  gene namely, *OsCIPK23*, in both development as well as abiotic stress response [66]. High expression of this gene was observed in anther and pistil, which was up regulated after pollination. Also, *OsCIPK23*  was induced in response to abiotic stress conditions such as cold, drought and salt. Induction of this gene was also observed after giberrellin and ABA treatment. Both RNAi as well as overexpression approach showed *OsCIPK2*3 to be essential for seed development and drought resistance. Subcellular localization using GFP constructs showed this kinase to be mainly a nuclear protein with weak expression in cytosol [66].

### CIPKs in other Plants

Study aiming to determine CIPK genes in rice also identified the CBL-CIPK gene network in other crop plant such as wheat (11 CBLs and 29 CIPKs), barley (9 CBLs and 14 CIPKs) and soybean (7 CBLs and 13 CIPKs) [71]. Besides these, other plants in which these genes were found to be present included alfalfa or *Medicago trunculata*  (9 CBLs and 11 CIPKs), Gymnosperm sp. *Pinus*  sp. (2 CBLs and 7 CIPKs) and moss *Physcomirtella patens*  (4 CBLs and 3 CIPKs) [71]. Genome wide analysis of Poplar (*Populus trichocarpa*), which is a woody plant with known genome sequence, has revealed the existence of 27 CIPKs in this tree species [89].

Studies on other plant species such as maize have also provided support for the existence of CBL-CIPK signaling pathway in these plants where expression of different CIPK members under stress conditions combined with other molecular genetic evidence and localization studies clearly establishes the importance of this plant specific CBL-CIPK signaling pathway in abiotic stress adaptation. ZmCIPK16 which shares high level of amino acid identity with OsCIPK16 (79%) and AtCIPK16 (51%) was found to be showing up regulation at the transcript level under drought, ABA, PEG, cold, salt and heat treatment [90]. RNA blots have indicated significant rise in the level of *ZmCIPK16*  in PEG treated root and shoot. Maize seedlings were found to be accumulating highest level of *ZmCIPK16*  transcripts after 12h of salt stress, 24h of heat stress, and after 6h in case of both ABA and drought treatment. However, no such rise was observed in the level of *ZmCIPK16*  after cold treatment. Expression of *ZmCIPK16*  in *sos2*  mutants of Arabidopsis partially restored the salt sensitive phenotype in these plants providing strong support for the involvement of *ZmCIPK16*  in tolerating salt stress. Also, expression of *sos1*  was enhanced in the transgenic plants overexpressing the *ZmCIPK16*, which showed that ZmCIPK16 was acting by directly activating the *AtSOS1*  expression. As an effort to map the interacting partners of CIPK16 from maize, yeast two hybrid studies were also performed, and ZmCIPK16 was found to be interacting with all the tested ZmCBLs, namely, ZmCBL3, 4, 5 and 8 at varying strength. Strongest interaction was observed between ZmCBL4 and ZmCIPK16 which perhaps indicate the role of both these interacting partners in the salt stress signaling as previous study has shown *ZmCBL4*  expression in Arabidopsis to be improving salt tolerance [91]. ZmCIPK16 was localized to the nucleus, cell membrane and to a lesser extent in the cytosol. The ZmCBL3, 4 and 5 were found to be restricted to the plasma membrane, and the complex of these maize CBLs with ZmCIPK16 in the BiFC assay were found to be plasma membrane localized. Of these, ZmCBL4 and 5 were determined to be having the N-myristoylation motif and ZmCBL3, which lacks this membrane-anchoring motif, is believed to be having several palmitoylation sites, which might aid in its localization to the membrane. Similar studies in rice have shown that OsCBL2 and OsCBL3, which do not have any such predicted membrane localization domains, are anchored to the tonoplast while OsCBL4 with a myristoylation motif is located at the plasma membrane [92]. All such localization signals anchoring the CIPK in specific cell compartments by CBLs is reflective of their site of function where the CBL-CIPK complex might be acting on other target proteins.

Study in Pea (*Pisum sativum*), which is a legume plant, has also shown the existence of CBL-CIPK signaling network, and its role in both abiotic and biotic stress conditions [93]. PsCIPK and PsCBL were induced under salt, cold and wounding stress, but no difference in expression was found upon dehydration or ABA treatment. Transcript accumulation for both PsCBL as well as PsCIPK was observed upon salicylic acid and calcium treatment. This might be due to the involvement of this CBL-CIPK combination in biotic stresses, as salicylic acid is commonly produced when plants face a pathogenic attack causing biotic stress. Immunolocalization experiments found PsCIPK to be both cytosol as well as membrane associated. Also, PsCIPK and PsCBL were found to be interacting in yeast two-hybrid analysis, which might reflect their actual interaction *in vivo*  that helps localize PsCIPK to the membrane. Two CBL and one CIPK genes were also identified in cotton (*Gossypium hirsutum*) that showed overlapping expression in the cotton fibers indicating their possible function in fiber elongation [94]. Selective interaction could be seen of GhCIPK1 with both GhCBL2 and GhCBL3 during yeast two-hybrid assay [94].

## OTHER CALCIUM REGULATED PROTEIN KINASES AND ABIOTIC STRESS

Besides CDPKs and CIPKs, there are other classes of calcium regulated kinases which are yet to be explored in detail, but are postulated to be playing an important role in abiotic stress signaling based on several reports. CDPK-related protein kinase or (CRK) and Ca^2+^/CaM kinase or (CCaMK) are two such classes of CDPK-related kinases that have been studied in different plant varieties. CRKs like CDPKs, possess a N-terminal myristoylation motif which might help in plasma membrane associations. However, CRKs have degenerate EF-hands that are incapable of binding calcium ions [23]. CDPK related protein kinase or CRKs have been studied in plant species such as maize and Arabidopsis. Almost all of the CRKs studied till date are found to be showing kinase activity that is both Ca^2+^ and CaM independent. AtCRK3 from Arabidopsis like ZmCRK from maize exhibits autophosphorylation and substrate phosphorylation that is independent of both Ca^2+^ and CaM [95, 96]. However, kinase activity of AtCRK3 was inhibited by 1mM of Ca^2+^. Although this is much higher than physiological concentration of calcium, it indicates a possible mechanism of calcium regulation of AtCRK3. *AtCRK3*  expression was induced by ABA as analyzed by northern hybridization. Since ABA is commonly produced under stress conditions, and induces cytosolic changes in Ca^2+^, it was proposed by the authors that AtCRK3 could possibly be participating in ABA stress signaling by phosphorylating specific substrates. Abundant transcript levels could be detected in vascular bundles of stem and leaf as well as in tapetum layer of pollen mother cells [95]. A characteristic differential expression pattern was observed during pollen development. The *AtCRK3*  transcripts gradually increased and showed highest expression at the tetrad stage, and then, declined below detection level at the mature pollen stage. Tapetum, which is responsible for providing nutrition to the growing pollen, also had a similar expression profile of AtCRK3 indicating that this kinase might be important for function and metabolism of the tapetal layer.

AtGLN1 (cytosolic Glutamine synthetase involved in nitrogen assimilation) has been identified as a potential substrate for AtCRK3 using yeast two-hybrid approach [97]. This interaction was also confirmed using co-immunoprecipitation assays. Both AtGLN1 and AtCRK3 were induced under early senescent stage, and AtCRK3 specifically phosphorylated AtGLN1. Such interaction is believed to be important for nitrogen mobilization during leaf senescence.

Identification and biochemical characterization of another CRK from Arabidopsis, AtCRK1, has shown this kinase to be binding to CaM isoforms in a calcium dependent manner [98]. Binding of the different CaM-isoforms, namely, AtCaM2, AtCaM4, AtCaM7 and AtCaM8 stimulated the kinase activity of AtCRK1 to similar extent. However, the autophosphorylation and substrate phosphorylation activities for this kinase were found to be calcium independent. Thus, different CRKs sharing high sequence identity (as in case of AtCRK1 and AtCRK3) could be regulated by diverse mechanisms specific to the protein kinase.

Calmodulin binding kinase (CaMK), and Calcium and calmodulin regulated kinase (CCaMK), are both calmodulin binding protein kinases (CBKs), which possess a CaM binding domain. CaMKs form a large family of enzymes in animals [14]. In contrast, very few members of this type of kinase have been reported in plants such as apple and tobacco [99, 100]. CCaMKs are structurally similar to CDPKs, however, these have three EF-hands which are similar to the C-terminal domain of brain protein visinin [23]. Autophosphorylation of CCaMK is promoted by binding of calcium, which further stimulates its association with Ca^2+^/CaM complex. This Ca^2+^/CaM complex is found to enhance substrate phosphorylation by CCaMK. Several reports have provided insight into the biochemical as well as expression based characteristics of CCaMK in different plant species such as carrot, apple, maize and lily [101]. However, no representative CaMK or CCaMK has been reported from Arabidopsis [14].

NtCBK2, is a CaM binding protein kinase which is stimulated specifically by GA and salt stress [102]. Of the different stages, developing anther, pistil and embryo showed high expression of this gene. AtCBK3 is also a Ca^2+^/CaM kinase that has been shown to be important in heat stress signaling. Yeast two-hybrid study found AtCBK3 to be interacting with AtHSF1a [103]. MCK1 and MCK2 are CRK and Ca^2+^/CaM kinase, respectively from maize that showed a temporal and spatial variation in the pattern of expression during development [104]. MCK1 was identified as a CCaMK [105] and later classified as a CRK based on sequence analysis [23].

*PsCCaMK*  from pea was seen to be involved in stress signaling [106] while *NtCaMK1*  is up regulated upon salt stress treatment [100]. Increased accumulation of *PsCCaMK*  was observed in root tissue upon low temperature and salinity stress [106]. Tissue specific expression studies have been conducted for CCaMK from lily and tobacco where lily CCaMK was restricted to pollen mother and tapetal cells [100]. Tobacco CCaMK1 and CCaMK2 show mRNA and protein expression specific to the meiosis stage of the anther development [100]. Despite several expression analyses studies of these calcium regulated kinases, and biochemical characterization for a few of them, not much has been elucidated regarding their detail *in plant*  function, and future studies are required to understand the exact molecular function performed by these proteins in signaling pathways.

## CONCLUSION AND FUTURE PERSPECTIVE

Calcium plays a pivotal role in multiple biological processes including signaling events mediated in abiotic stress in plants. Calcium sensors are the proteins, which sense the “calcium signature” and transduce the signal downstream through a number of protein components. In plants, there are three major classes of calcium signaling kinases, which exist as multigene families. Gene expression profiling at single and whole genome level have provided a crucial biological clue for deciphering the function of these genes at different developmental and environmental conditions. Expression analysis for these calcium signaling kinases at both transcript (transcriptomic) and protein (proteomic) level has caught attention of several leading research groups who are now attempting to determine, and integrate this important information for investigating the functional analysis of these genes or proteins families.

Multiple approaches are required to understand the functions of these proteins that include site-directed mutagenesis or deletion of key residues or domains, reverse genetics (knockouts) to identify null mutations, overexpression studies, protein interaction screens to identify potential substrates, biochemical analyses to characterize kinetic properties, and integration of expression and localization studies to clarify where and when various family members are expressed. Existence of multigene family for these calcium-signaling kinases that are redundant in function poses a major challenge for functionally characterizing these by reverse genetic, overexpression studies, and other molecular and biochemical approaches. Therefore, a detail expression profiling of these families under different developmental and stress conditions will provide crucial biological information to plan an in-depth molecular dissection of individual and multiple genes in plants. Moreover, a comprehensive identification of downstream targets of these kinases is required in order to understand the mechanistic details of a given signaling pathway. In addition, a major challenge for the future research is the elucidation of the interconnections and synergistic functions of these gene families in the diverse signaling network in plants. The cross talk of this calcium signaling kinases with other signaling pathways such as receptor like-kinase (RLKs), two-component systems, and mitogen associated protein kinases (MAPK) cascades also require further investigation. Ultimately, utilizing the tools of transgenic technology and molecular breeding to enhance abiotic stress tolerance by employing key components of these calcium-signaling kinases in agriculturally important crop needs to be undertaken.
